# Utilizing a Community-Based Partnership to Increase Health Promotion Among Rural Older Adults

**DOI:** 10.1093/geroni/igaa057.1861

**Published:** 2020-12-16

**Authors:** Cassandra Ford, Martha Crowther, Keri Barron, Mary Ann Kelley

**Affiliations:** 1 The University of Alabama, Tuscaloosa, Alabama, United States; 2 The University of Alabama Capstone College of Nursing, Tuscaloosa, Alabama, United States

## Abstract

Older adults living in rural communities often encounter health disparities related to chronic conditions and access to care. In an effort to address these disparities, a community-based program focused on health promotion and education was implemented with community dwelling, rural, African American older adults. We partnered with a local church to provide education regarding health promotion, chronic disease self-management, and support for senior group members (n=32). Program materials were accessed online in order to facilitate sustainability and potential program expansion. Key findings from a qualitative content analysis indicated the majority of participants reported improved engagement in chronic disease management and health promotion activities (i.e., following a healthy diet, monitoring blood pressure, taking medications as prescribed), incorporating health behaviors to prevent the development of co-morbid conditions, and increased client-provider communication. Implications for further research will be discussed and key elements of program implementation such as a continued partnership will be explored.

